# Effects of L‐Carnitine and Fenofibrate on Lipid Metabolism, Oxidative Status, and Flesh Quality of Yellow River Carp (*Cyprinus carpio*)

**DOI:** 10.1155/anu/1500743

**Published:** 2026-08-11

**Authors:** Mengjuan Zhao, Shaoyang Zhi, Chaobin Qin, Liping Yang, Guoxing Nie

**Affiliations:** ^1^ Aquatic Animal Nutrition and Feed Research Laboratory, College of Fisheries, Henan Normal University, Xinxiang 453007, China, henannu.edu.cn

**Keywords:** fenofibrate, L-carnitine, lipid metabolism, muscle quality, oxidative stress, Yellow River carp

## Abstract

This study aimed to investigate the regulatory effects of dietary supplementation with L‐carnitine and fenofibrate under high‐fat (HF) diet conditions. Yellow River carp (*Cyprinus carpio*; initial body weight 156 g) were assigned to control (CO), HF, L‐carnitine supplements at 200 and 500 mg/kg (L200 and L500), and fenofibrate supplements at 200 and 500 mg/kg (F200 and F500). Each group consisted of three cages and was reared for 8 and 12 weeks to assess both short‐term and long‐term responses. At 8 weeks, compared to the CO group, the HF group showed a higher weight gain rate (WGR), intraperitoneal fat index (IFI), serum triglyceride (TG), intramuscular fat (IMF), and elevated hepatopancreas malondialdehyde (MDA) and alanine aminotransferase (ALT). At 12 weeks, only WGR decreased slightly compared to 8 weeks, whereas IMF, IFI, and MDA remained higher than the CO group (*p*  < 0.05). Both L‐carnitine and fenofibrate reduced IMF at 8 and 12 weeks and increased muscle hardness, chewiness, and gumminess. Additionally, the L500 increased the muscle glutathione peroxidase (GSH‐Px) at 8 weeks, and the F200 increased the GSH‐Px at 12 weeks (*p*  < 0.05). Muscle fiber analysis showed reduced diameter and increased density in the L200 group at 8 weeks and increased fiber density in the F500 group at 12 weeks. At the molecular level, both supplementations upregulated muscle peroxisome proliferator–activated receptor γ coactivator 1‐α (*pgc-1α*), 5′‐AMP‐activated protein kinase alpha 2 (*ampkα2*), and carnitine acyl transferase 1 (*cpt-1*), while lipogenic gene fatty acid synthase (*fas*) was downregulated in the F200 and F500 groups, and sterol regulatory element‐binding protein 1 (*srebp1*) was reduced in the F500 group (*p* < 0.05). At 12 weeks, *fas* remained downregulated in all supplementation groups, and *pgc-1α* remained upregulated in the L200 group, whereas *cpt-1*, *ampkα2*, and *pgc-1α* were increased in the F200 group (*p* < 0.05). Overall, L‐carnitine and fenofibrate effectively mitigate HF‐induced lipid overaccumulation and oxidative stress, thereby enhancing muscle structural integrity and nutritional value in Yellow River carp.

## 1. Introduction

Aquatic products are a crucial source of necessary fatty acids and higher‐quality proteins, which help maintain human nutritional health and global food security [1]. Due to their high energy density, protein‐sparing properties, and decreased nitrogen release into aquatic habitats, high‐fat (HF) diets have become more frequent in commercial production as intensive aquaculture has developed quickly [2]. However, prolonged intake of HF diets often disrupts lipid metabolic homeostasis in fish, leading to excessive lipid deposition, enhanced oxidative stress, and deterioration of flesh quality. These adverse effects pose a major challenge to the sustainable expansion of contemporary aquaculture since they not only impair fish health but also reduce the product value and production efficiency [3].

Muscle quality is a vital factor in determining the market competitiveness and consumer acceptance of cultured fish. Among various flesh quality traits, muscle texture, particularly hardness, is a critical sensory attribute that is closely associated with muscle fiber characteristics, collagen content, and intramuscular fat (IMF) deposition [4, 5]. IMF is primarily made up of triglyceride (TG), with lesser proportions of phospholipids and cholesterol, and is distributed between muscle fibers and myocytes [6]. Although moderate IMF contributes to improved juiciness and flavor, excessive IMF accumulation induced by HF feeding often leads to loosened muscle structure, reduced hardness, and impaired elasticity. Evidence from Atlantic salmon (*Salmo salar*) [7], Nile tilapia (*Oreochromis niloticus*) [8], and grass carp (*Ctenopharyngodon idella*) [9] consistently demonstrates that HF‐induced lipid overload often results in oxidative stress and lipid peroxidation, ultimately resulting in muscle softening and deterioration of flesh quality. Therefore, improving the quality of cultured fish requires nutritional strategies that precisely regulate muscle lipid deposition while reducing oxidative stress.

Dietary feed additives represent an efficient and practical approach for targeted nutritional regulation in aquaculture systems [10]. Among them, L‐carnitine and fenofibrate are two representative functional additives with distinct regulatory mechanisms. L‐carnitine is widely considered a security feed additive because it is essential for long‐chain fatty acids to be able to access the mitochondria for β‐oxidation, which enhances lipid utilization and energy metabolism [11]. According to earlier research, L‐carnitine effectively reduces lipid accumulation in the hepatopancreas and muscle of black seabream (*Acanthopagrus schlegelii*) [12], largemouth bass (*Micropterus salmoides*) [13], and tilapia [14] while improving antioxidant capacity and muscle quality. Fenofibrate, a widely recognized agonist of peroxisome proliferator–activated receptor alpha (PPARα), activates lipid metabolism–related downstream signaling pathways, thereby suppressing TG synthesis [15]. Its lipid‐lowering and antioxidant effects have been documented in several fish species, including Nile tilapia [16], yellow catfish (*Pelteobagrus fulvidraco*) [17], largemouth bass [18], and obscure puffer (*Takifugu obscurus*) [19]. Nevertheless, existing studies have largely focused on hepatic lipid metabolism, and systematic investigations into how these additives differentially regulate muscle lipid metabolism and flesh quality under HF feeding conditions remain limited.

The Yellow River carp is a primary species for freshwater aquaculture in northern China due to its quick growth, great environmental adaptability, and wide consumer acceptance. In recent years, however, intensive culture practices have increasingly resulted in excessive lipid accumulation and deteriorating flesh quality, which have become a major obstacle restricting the growth of the sustainable industry in Yellow River carp. Therefore, the current study set out to systematically assess the effects of dietary L‐carnitine and fenofibrate supplementation on growth performance, muscle quality, and lipid metabolic regulation in Yellow River carp fed a HF diet, supplying both theoretical insight and useful guidance for nutritional strategies to improve muscle quality and metabolic health in intensive carp aquaculture.

## 2. Materials and Methods

### 2.1. Ethical Statement

The total fish–related research was approved by Henan Normal University’s Experimental Animal Ethics Committee and was performed strictly in accordance with the guidelines for the use and care of laboratory animals (Document Number HNSD‐2024BS‐0409).

### 2.2. Experimental Diets

L‐carnitine and fenofibrate (purity ≥ 99%) were purchased from Shanghai Yuanye Bio‐Technology Co., Ltd. (Shanghai, China). The dietary ingredients were purchased from Minzhifeng Feed Sales Department (Zhengzhou, China), and the vitamin premix and mineral premix were purchased from Weinuo Breeding Co., Ltd. (Zhuhai City, China). Six isonitrogenous experimental diets were prepared (Table 1), comprising a control (CO) diet, 10% soybean oil in a HF diet, and four HF diets supplemented with 200 or 500 mg/kg L‐carnitine (L200 and L500) or fenofibrate (F200 and F500), respectively. Each component was thoroughly mixed after going through a 60‐mesh filter. Oils and water were subsequently added, and mixtures were pelleted, let to air dry at ambient temperature, and then kept at −20°C until use.

**Table 1:** Ingredient composition and proximate composition of experimental diets in this study (% dry matter).

Ingredients	Diets (%)					
	CO	HF	L200	L500	F200	F500
Fish meal	7	7	7	7	7	7
Wheat middling	8	8	8	8	8	8
Corn starch	10	10	10	10	10	10
Corn gluten starch	14	14	14	14	14	14
Soybean meal	32	32	32	32	32	32
Rapeseed meal	10	10	10	10	10	10
Soybean oil	3	10	10	10	10	10
Ca(H_2_PO_4_)_2_	2	2	2	2	2	2
Methionine	0.1	0.1	0.1	0.1	0.1	0.1
Lysine	0.3	0.3	0.3	0.3	0.3	0.3
Vitamin premix^a^	0.07	0.07	0.07	0.07	0.07	0.07
Mineral premix^b^	0.11	0.11	0.11	0.11	0.11	0.11
Choline chloride	1.75	1.75	1.75	1.75	1.75	1.75
Carboxymethyl cellulose	1.75	1.75	1.75	1.75	1.75	1.75
Microcrystalline cellulose	9.92	2.92	2.90	2.87	2.90	2.87
L‐carnitine	0	0	0.02	0.05	0	0
Fenofibrate	0	0	0	0	0.02	0.05
Total	100	100	100	100	100	100
Proximate composition (%)						
Moisture	90.17	90.58	90.12	90.28	90.37	90.88
Crude lipid	5.16	10.58	10.31	10.43	10.32	10.17
Crude protein	36.52	36.27	35.48	35.34	35.86	36.51

^a^Vitamin premix (g kg^−1^ premix): retinyl acetate, 1.21; cholecalciferol, 1.20; all‐rac‐a‐tocopherol, 20.00; menadione, 9.09; thiamine, 10.87; riboflavin, 7.50; ascorbic acid, 30.00; pyridoxine hydrochloride, 12.12; cyanocobalamin, 2.00; folic acid, 40.00; biotin, 12.50; nicotinic acid, 40.40; D‐capantothenate, 16.13; inositol, 204.08; cellulose, 592.89.

^b^Mineral premix (g kg^−1^ premix): FeC_6_H_5_O_7_, 11.43; ZnSO_4_⋅7H_2_O, 11.79; MnSO_4_⋅H_2_O (99 %), 2.49; CuSO_4_⋅5H_2_O (99 %), 1.06; MgSO_4_⋅7H_2_O (99 %), 27.31; KH_2_PO_4_, 233.2; NaH_2_PO_4_, 228.39; C_6_H_10_CaO_6_⋅5H_2_O (98 %), 34.09; CoCl_2_⋅6H_2_O (99 %), 1.36.

### 2.3. Experimental Fish and Rearing Conditions

A commercial aquaculture farm in Yanjin, Xinxiang, Henan Province, China, provided the healthy Yellow River carp. The feeding experiment was carried out at Henan Normal University’s Aquatic Animal Nutrition R&D and Pilot Testing Base in Luohe, China. The total of 18 net cages containing three replicates per group and 38 fish per cage were chosen at random from the entire 684 healthy fish with a homogenous genetic background and a normal initial body weight of 156 g. Throughout the entire experiment, water temperature was kept at 25–30°C, dissolved oxygen at ~8.8 mg/L, pH at 7.5 ± 0.4, and ammonia nitrogen less than 0.3 mg/L. Fish used for experiments were hand‐fed three times a day at 08:00, 12:00, and 17:00, with a standard feed ration of 3% of their body weight; the feed amount was adjusted once a week based on estimated body weight. Feeding activity and health status were monitored daily. The natural photoperiod was maintained.

### 2.4. Sampling Procedures

At weeks 8 and 12, the experimental fish were initially fasted for 24 h and then anesthetized with MS‐222 (55 mg/L) for collecting biological samples. Twelve fish were randomly selected from each cage, and ~1 mL of blood was collected from the caudal vein using a sterile syringe. After allowing the blood to clot at 4°C for 6 h, the serum was separated by centrifugation at 3500 g for 10 min and kept at −80°C for biochemical analysis. Following blood collection, muscle and hepatopancreas tissues were promptly flash‐frozen in liquid nitrogen and stored at −80°C for enzyme activity assays and RNA extraction. Additionally, three fish were randomly selected from each cage; the dorsal axial muscle samples were fixed in 4% paraformaldehyde (Servicebio, China) for histological staining; and the remaining muscle and hepatopancreas tissues were collected and stored at −40°C for conventional nutritional component analysis. Furthermore, muscle samples located between the dorsal fin and the lateral line were taken from the three randomly selected fish in each cage to evaluate muscle texture characteristics.

The calculation formulas for growth performance‐related indicators are as follows:

Weight gain rateWGR,%=final body weight– initial body weight/initial body weight×100,

Feed conversion ratioFCR,%=feed intake/final body weight– initial body weight×100,

Specific growth rateSGR,%/days=lnfinal body weight– lninitial body weight/days×100,

Viscerosomatic indexVSI,%=viscera weight/final body weight×100,

Hepatosomatic indexHSI,%=hepatopancreas weight/final body weight×100,

Intraperitoneal fat indexIFI,%=intraperitoneal fat weight/final body weight×100,

Condition factorCF, g/cm3=final body weight/ body length3×100,

Survival rateSR,%=final number of fish/initial number of fish×100.

### 2.5. Proximate Composition Analysis

The Official Methods of Analysis of AOAC international standard procedures (AOAC, 2006) were utilized to find out the proximate composition of samples. Samples were dried at 105°C to constant weight in order to assess the content of moisture. The Kjeldahl method was utilized to evaluate the content of crude protein. The Soxhlet extraction with petroleum ether was applied to estimate the content of crude lipid, and a muffle furnace was used to incinerate the ash at 550°C.

### 2.6. Histological Analyses

For H&E staining, fixed muscle samples were dehydrated with graded ethanol, cleared with xylene, and embedded in paraffin. The standard hematoxylin‐eosin staining technique was applied to 8 μm sections. Muscle fiber diameter and density were quantified using ImageJ software.

For Oil Red O staining, muscle samples were cryoprotected in 10%, 20%, and 30% sucrose solutions, embedded at −25°C, sectioned at 10 μm, and stained according to standard protocols. A light microscope was used to photograph and visualize the lipid droplet accumulation.

### 2.7. Biochemical Analyses

Commercial assay kits (Nanjing Jiancheng Bioengineering Institute, Nanjing, China) were applied to examine the contents of TG (A110‐2‐1), total cholesterol (T‐CHO, A111‐2‐1), high‐density lipoprotein cholesterol (HDL, A112‐2‐1), low‐density lipoprotein cholesterol (LDL, A113‐2‐1), total antioxidant capacity (T‐AOC, A015‐1‐2), superoxide dismutase (SOD, A001‐1‐2), malondialdehyde (MDA) (A003‐1‐2), aspartate aminotransferase (AST, C010‐1‐1), alanine aminotransferase (ALT) (C009‐1‐1), lactate dehydrogenase (LD, A019‐2‐1), hydrogen peroxide (H_2_O_2_, A064‐1‐1), hydroxyproline (HYP, A030‐2‐1), and glutathione peroxidase (GSH‐Px) (A005‐1‐2). The BCA protein assay kit (Solarbio, PC0020) was utilized to assess the protein concentration.

### 2.8. Muscle Texture Analysis

Texture profile analysis was performed on dorsal muscle samples from nine fish per group. From each fish, scales were carefully removed from both sides of the body between the dorsal fin and the lateral line (avoiding skin damage), and four pieces of dorsal muscle tissue (2 cm × 2 cm × 1 cm) were then collected from each side for analysis. A texture analyzer (Brookfield, Middleboro, USA) was applied to analyze both raw and cooked flesh meat. The instrument was calibrated at a strain rate of 60% and a 5 mm/s test speed. Shear force, hardness, chewiness, springiness, gumminess, and resilience were among the texture parameters that were recorded.

### 2.9. Fatty Acid Composition Analysis

Samples of freeze‐dried muscle were treated with a chloroform–methanol mixture (2:1, v/v) to extract total lipids. 2.5% H_2_SO_4_ in methanol was used to methylate fatty acids for 2 h at 70°C. Following filtering over a 0.22 μm membrane, fatty acids were extracted and determined through gas chromatography. The fatty acid composition was determined according to the fatty acid standard of Sigma and calculated by the area normalization method: gas chromatography conditions: The gas chromatography column was a DB‐WAX column of Hewlett–Packard (15 m × 250 μm × 0.25 μm); column oven temperature conditions: Initial temperature of column oven was 120°C, and maximum temperature was 250°C; injection port settings: Heater temperature was 250°C, carrier gas was high‐purity nitrogen, flow rate was 1.4 mL/min, and split ratio was 10:1; and detector conditions: Heater temperature was 250°C, air flow rate was 400 mL/min, hydrogen gas flow rate was 40 mL/min, and tail gas nitrogen flow rate was 25 mL/min. The specific operation steps can be found in method described by Xie et al.

### 2.10. RNA Extraction and Gene Expression (*gpx*) Analysis

The TRIzol reagent (Takara, Japan) was utilized to obtain total RNA from the muscle tissue. A NanoDrop 2000 spectrophotometer (Thermo, USA) was adopted to measure the concentration and purity of RNA, and agarose gel electrophoresis was used to confirm its integrity. The PrimeScript RT reagent kit with gDNA Eraser (Takara, Japan) was chosen to create first‐strand cDNA. SYBR Premix Ex Taq II (Vazyme, China) was applied for qPCR on a Roche LightCycler 480 equipment. Each reaction contained 2.5 μL SYBR mix, 0.25 μL of each primer, 3.5 μL DEPC‐treated water, and 1 μL cDNA. The reference gene was 18S rRNA, and the 2^–ΔΔCt^ method was employed to calculate the relative expression. Primer efficiencies were close to 100%, and primer sequences are displayed in Table 2.

**Table 2:** Real‐time PCR primer sequences.

Gene	Sequence (5′–3′)	GenBank ID
*fas* F	GACAGGCCGCTATTGCTATT	GQ466045.1
*fas* R	TGCCGTAAGCTGAGGAAATC	
*acc* F	GTCACTGGCGTATGAGGATATT	XM_042757417.1
*acc* R	TCCACCTGTATGGTTCTTTGG	
*srebp* F	CGTCTGCTTCACTTCACTACTC	XM_042730308.1
*srebp* R	GGACCAGTCTTCATCCACAAA	
*lpl* F	CGCTCCATTCACCTGTTCAT	FJ716101.1
*lpl* R	GCTGAGACACATGCCCTTATT	
*cpt-1* F	CAGATGGAAAGTGTTGCTAATGAC	JQ361077.1
*cpt-1* R	TGTGTAGAAGTTGCTGTTGACCA	
*pparα* F	GCGTGCTTTGGCTTTGTT	FJ849065.1
*pparα* R	GGGAAAGAGCAGCACGAG	
*ampkα1* F	TGCGAGAAGTTTGAGTGCAC	PQ804663
*ampkα1* R	TTCATAATGCGCCGGTTGTC	
*ampkα2* F	AAATGTGTGCCAGCCTCATC	PQ804664
*ampkα2* R	ATGTTTGTGGCGCTACTGTG	
*elovl5a* F	GAGGATGGCTTCTACTG	MK893918.1
*elovl5a* R	ATCTGCCTTGATACACT	
*elovl5b* F	TCGGGTTAGAGGATGGC	MK893919.2
*elovl5b* R	CTGGCAGAAGAAGTTGTAT	
*pgc-1α* F	TGCTGCTTTGGTTGGTGAAG	AB767302.1
*pgc-1α* R	TGCATCGAGGTCACTGACATC	
SOD F	GTCCGCACTTCAACCCTCAT	XM 019111527.2
SOD R	ATGGTCCTCCCAATGACCGA	
*cat* F	TTCCTGTGGGACGCCTTGT	JF411604.1
*cat* R	TCCGAGCCGATGCCTATGT	
*gpx1* F	AACCAGTTCGGACATCA	GQ376155.1
*gpx1* R	ATCACCCATCAAGGACA	
*keap1* F	CAGTGGGCGAGAAGTGT	JX470752.1
*keap1* R	TTTGATGGCTCCAGGTT	
*nrf2* F	ACGACAAATGCCGAAGT	JX462955.1
*nrf2* R	CTGCCTCATCTAGTGGAAA	
*tgf-β* F	GCTACCTGGAATCACGCTTTA	XM_019072371.2
*tgf-β* R	CTTGCTCTGCCTCACTTCTC	
*smad2* F	GCTACCCTGACCAAACCA	XM_019079971
*smad2* R	GACGCAGACTTCATCCTT	
*smad4* F	CAGGTGGCTGGTCGTAAA	XM_019122985
*smad4* R	CGTAATGGTAAGGGTTCA	
*pax7* F	GCTCCATTAGTCGGGTTC	XM_042766854.1
*pax7* R	GGCTCCGACTCCACATC	
*myod* F	CAACGACACGCCAAAT	XM_019068329.2
*myod* R	CTGACAGCACGGGACA	
*myog* F	GAAGGCGGCGATAACTTC	XM_019096789.2
*myog* R	CTGCTGCTCCTGGTGAG	
*mrf4* F	TGTCTTATGTGGGCTTGT	XM_019092292.2
*mrf4* R	CTCTGGTTCGGATTGG	
*mylc* F	ACAGAACCCAACCAACA	XM_042731729.1
*mylc* R	GAATACACGCAGACCCT	
*myhc* F	TGAACCCTCTGTGCTGT	XM_042724157.1
*myhc* R	CTCCATACGCTTCTTGC	
*pcna* F	GACAAGGAGGATGAAGCG	XM_019123355
*pcna* R	TTTGGACAGAGGAGTGGC	
*18s* F	GAGACTCCGGCTTGCTAAAT	FJ710826.1
*18s* R	CAGACCTGTTATTGCTCCATCT	

*Note: 18s*, reference gene.

Abbreviations: *acc*, acetyl‐coA carboxylase; *ampkα1*, 5′‐AMP‐activated protein kinase alpha1; *ampkα2*, 5′‐AMP‐activated protein kinase alpha 2; *cat*, catalase; *cpt-1*, carnitine acyl transferase 1; *elovl5a*, elongation of very long chain fatty acids protein 5a; *elovl5b*, elongation of very long chain fatty acids protein 5b; *fas*, fatty acid synthase; *gpx*, glutathione peroxidase; *keap1*, kelch‐like ECH‐associated protein 1; *lpl*, lipoprotein lipase; *mrf4*, myogenic regulatory factors 4; *myhc*, myosin heavy chain; *mylc*, myosin light chain; *myod*, myogenic differentiation; *myog*, myogenin; *nrf2*, nuclear factor erythroid 2–related factor 2; *pax7*, paired box 7; *pcna*, proliferating cell nuclear antigen; *pgc-1*α, peroxisome proliferator–activated receptor γ coactivator 1‐α; *pparα*, peroxisome proliferator–activated receptor alpha; *smad2*, smad family member 2; *smad4*, smad family member 4; SOD, superoxide dismutase; *srebp1*, sterol regulatory element–binding protein 1; *tgf-β*, transforming growth factor‐β.

### 2.11. Statistical Analysis

The experimental results were displayed as mean ± standard error of the mean (SEM) after all data were examined utilizing SPSS 25.0 (IBM Corp., USA). Significant differences among dietary treatments are indicated by different letters, as evaluated by one‐way ANOVA, followed by Duncan’s test. A significance level of *p*  < 0.05 was considered statistical.

## 3. Results

### 3.1. Effects of Dietary Treatments on the Growth Performance of Yellow River Carp

**Figure 1:**
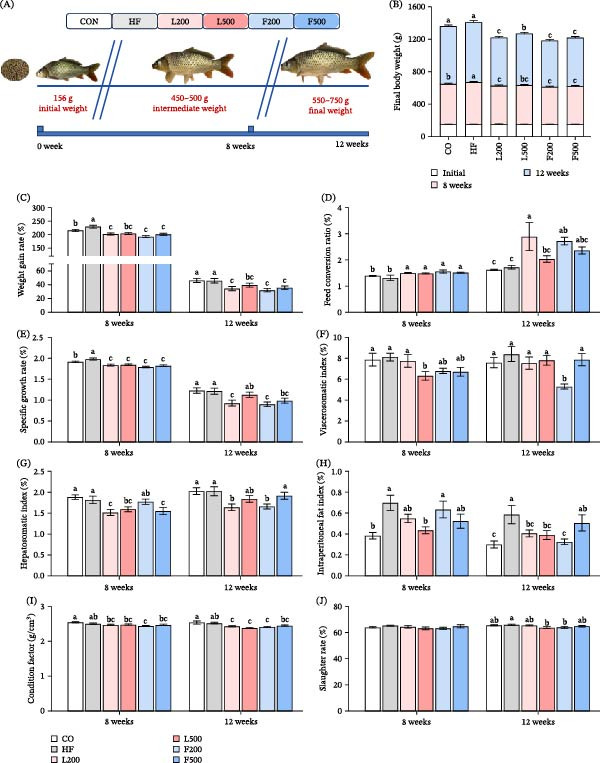
Growth performance and biological parameters of Yellow River carp (*Cyprinus carpio*) fed experimental diets. (A) Experimental grouping and temporal design for the feeding trial. (B) Final body weight (FBW). (C) Weight gain (WGR). (D) Feed conversion ratio (FCR). (E) Specific growth rate (SGR). (F) Viscerosomatic index (VSI). (G) Hepatosomatic index (HSI). (H) Intraperitoneal fat index (IFI). (I) Condition factor (CF). (J) Slaughter rates (SRs). Values are presented as means ± SEM from triplicate tanks (*n* = 9). Different letters indicate significant differences among dietary treatments as determined by one‐way ANOVA followed by Duncan’s test (*p*  < 0.05).

Growth performance and biometric indicators are presented in Figure 1. At 8 weeks, the HF group exhibited clearly higher final body weight (FBW), weight gain rate (WGR), and specific growth rate (SGR) than the CO group (*p*  < 0.05). In contrast, FBW and WGR in the L200 and F200 groups were significantly lower than in the HF group (*p*  < 0.001). The HF and CO groups had a noticeably lower feed conversion ratio (FCR) than the additive‐supplemented groups (*p*  < 0.05).

At 12 weeks, the HF group still showed the highest FBW and SGR, whereas L200 and F200 remained the lowest (*p*  < 0.05). Notably, the L200 group showed a high FCR compared to the HF and CO groups (*p*  < 0.05).

At both week 8 and week 12, intraperitoneal fat index (IFI) was clearly increased in the HF group (*p* < 0.05). Hepatosomatic index (HSI) was obviously lower in the L500 group at 8 weeks and in the F200 group at 12 weeks in comparison to the HF group (*p*  < 0.05).

### 3.2. Effects of Dietary Treatments on Serum Biochemical Parameters of Yellow River Carp

**Figure 2:**
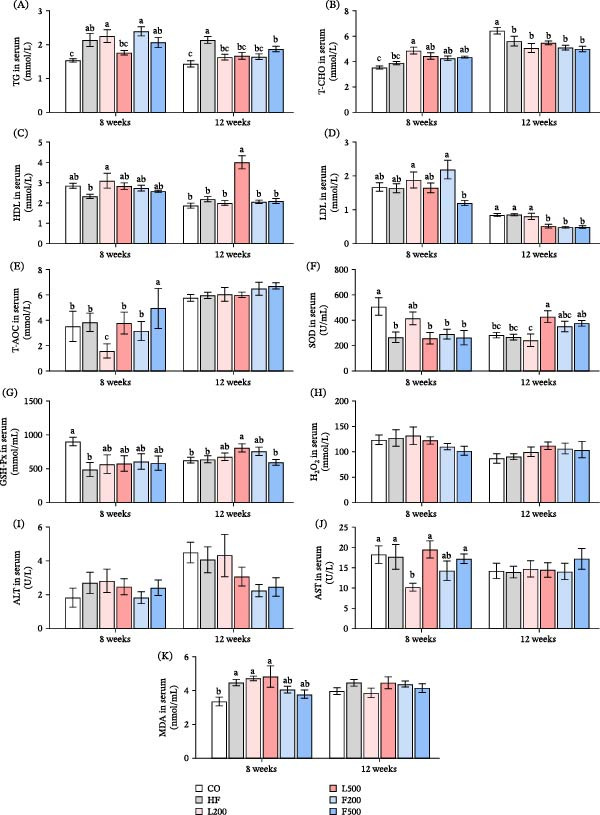
Serum biochemical parameters of Yellow River carp (*Cyprinus carpio*) fed experimental diets. (A) Triglyceride (TG). (B) Total cholesterol (T‐CHO). (C) High‐density lipoprotein (HDL). (D) Low‐density lipoprotein (LDL). (E) Total antioxidant capacity (T‐AOC). (F) Superoxide dismutase (SOD). (G) Glutathione peroxidase (GSH‐Px). (H) Hydrogen peroxide (H_2_O_2_). (I) Alanine aminotransferase (ALT). (J) Aspartate aminotransferase (AST). (K) Malondialdehyde (MDA). Values are means ± SEM from triplicate tanks (*n* = 9) at 8 and 12 weeks. The different letters indicate significant differences among dietary treatments as determined by one‐way ANOVA followed by Duncan’s test (*p*  < 0.05).

Serum biochemical parameters are shown in Figure 2. At both 8 and 12 weeks, TG content in the HF group was clearly higher than in the CO group (*p*  < 0.05). At 12 weeks, dietary supplementation with L‐carnitine or fenofibrate considerably reduced TG and LDL content compared with the HF group (*p*  < 0.05). Regarding antioxidant capacity, serum SOD and GSH‐Px activities in the HF group were much lower than in the CO group at 8 weeks (*p*  < 0.05). At 12 weeks, the L500 group had clearly higher SOD and GSH‐Px activities than the HF group (*p*  < 0.05).

### 3.3. Effects of Dietary Treatments on Muscle Histology and Lipid Deposition in Yellow River Carp

Muscle fiber characteristics are shown in Figure 3. At 8 weeks, muscle fiber diameter was significantly smaller and fiber density was notably elevated in the L200 group than in the CO group (*p*  < 0.05). At 12 weeks, the F500 group had a clearly higher fiber density than the HF group (*p*  < 0.05).

Figure 3Muscle fiber characteristics of Yellow River carp (*Cyprinus carpio*) fed experimental diets. (A, B) H&E staining of muscle at 8 and 12 weeks, respectively. (C, D) Distribution of muscle fiber diameters at 8 and 12 weeks, respectively. (E) Quantification of muscle fiber density. (F) Quantification of muscle fiber diameter based on H&E‐stained sections. Values are presented as means ± SEM from triplicate tanks (*n* = 6). The different letters indicate significant differences among dietary treatments as determined by one‐way ANOVA followed by Duncan’s test (*p*  < 0.05).

**Figure:**
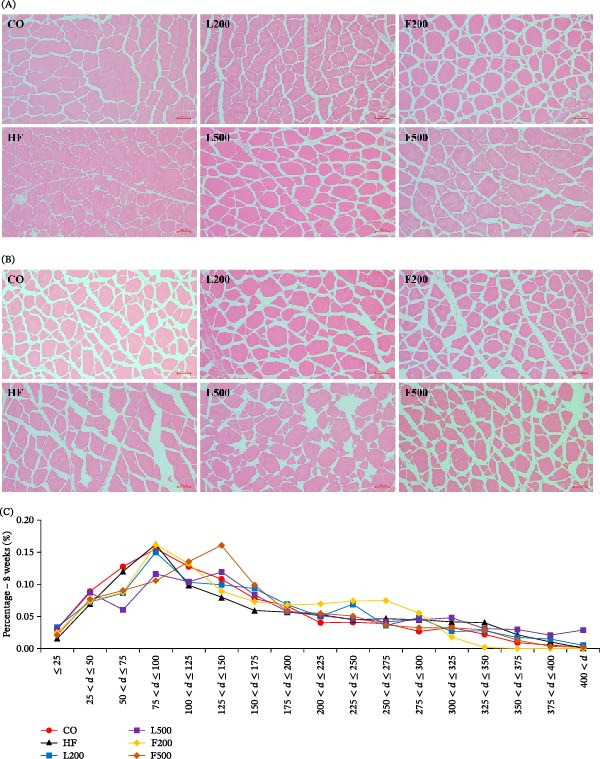

**Figure:**
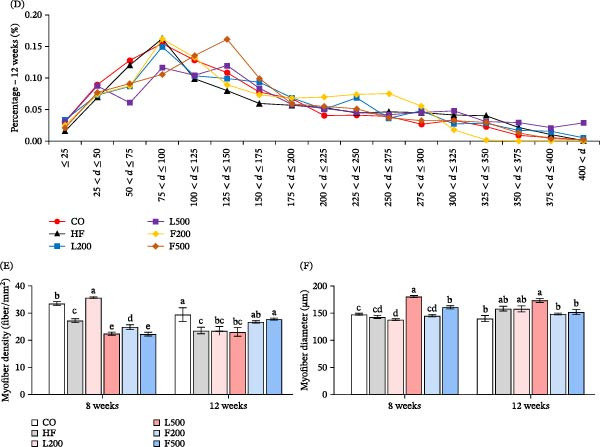

**Figure 4:**
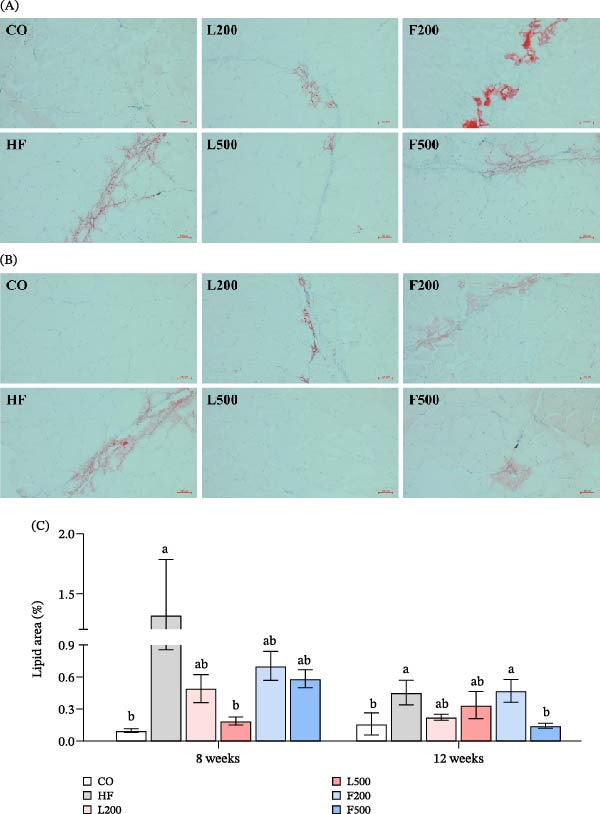
Oil Red O staining of muscle in Yellow River carp (*Cyprinus carpio*) fed experimental diets. (A, B) Representative Oil Red O–stained muscle sections at 8 and 12 weeks, respectively. (C) Quantification of lipid droplet area in muscle. Values are means ± SEM from triplicate tanks (*n* = 9) at 8 and 12 weeks. The different letters indicate significant differences among dietary treatments as determined by one‐way ANOVA followed by Duncan’s test (*p*  < 0.05).

Oil Red O staining results are shown in Figure 4. At 8 weeks, the lipid droplet area was substantially higher in the HF group than in the CO group (*p*  < 0.05), whereas it was significantly reduced in the L500 group compared with the HF group (*p*  < 0.05). At 12 weeks, lipid droplet areas were generally lower than those at 8 weeks across treatments. The HF group still remained noticeably higher lipid droplet area than the CO group, while the F500 group showed an obvious reduction relative to the HF group (*p*  < 0.05).

### 3.4. Effects of Dietary Treatments on the Muscle Texture Properties of Yellow River Carp

**Figure 5:**
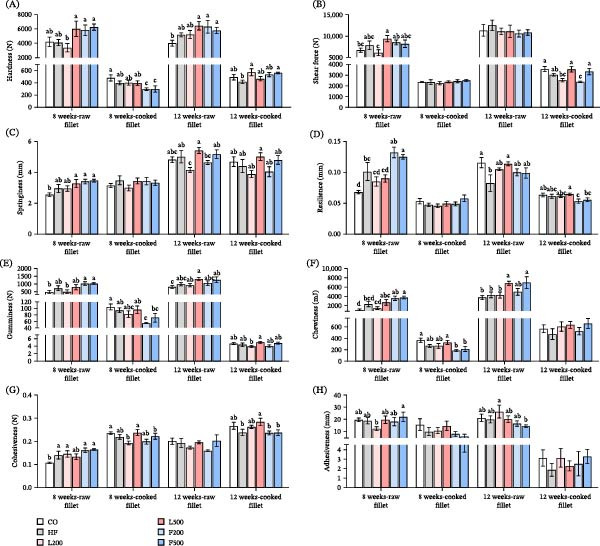
(A) Hardness, (B) shear force, (C) springiness, (D) resilience, (E) gumminess, (F) chewiness, (G) cohesiveness, and (H) adhesiveness.

Texture profile assessment results are exhibited in Figure 5. At 8 weeks, the F200 and F500 groups exhibited significantly improved texture properties of raw fillets, including hardness, chewiness, springiness, gumminess, and cohesiveness, relative to the CO group (*p*  < 0.05). In addition, the L500 group showed clearly higher springiness, shear force, and chewiness than the CO group (*p*  < 0.05).

At 12 weeks, the hardness, chewiness, and gumminess of raw fillets in the F500 and L500 groups remained considerably higher than in the HF group (*p*  < 0.05). No discernible changes were observed among dietary treatments in texture parameters of cooked fillets (*p*  > 0.05).

### 3.5. Effects of Dietary Treatments on the Muscle Proximate Components of Yellow River Carp

Whole‐body composition is displayed in Table 3. At 8 weeks, the HF group had considerably more crude lipid content in muscle than any other group, whereas moisture content was much lower (*p*  < 0.05). Supplementation with L‐carnitine or fenofibrate significantly reduced muscle lipid content as compared to the HF group (*p*  < 0.05). At 12 weeks, the HF group remained the highest in muscle lipid content, while all supplemented groups showed significantly reduced lipid levels relative to HF (*p*  < 0.001).

**Table 3:** Proximate composition of muscle in Yellow River carp (*Cyprinus carpio*) fed experimental diets.

Index	CO	HF	L200	L500	F200	F500	SEM	*p*‐Value
Muscle‐8 weeks (%)								
Moisture	79.65 ± 0.08^a^	79.01 ± 0.20^c^	79.41 ± 0.09^ab^	79.67 ± 0.07^a^	79.12 ± 0.13^bc^	79.46 ± 0.09^ab^	0.066	0.001
Crude lipid	0.91 ± 0.06^b^	1.16 ± 0.08^a^	0.96 ± 0.05^b^	0.95 ± 0.04^b^	0.95 ± 0.06^b^	0.91 ± 0.03^b^	0.056	0.041
Crude protein	18.57 ± 0.07^bc^	19.07 ± 0.12^a^	18.44 ± 0.03^c^	18.34 ± 0.12^c^	18.86 ± 0.13^ab^	18.58 ± 0.14^bc^	0.020	0.001
Muscle‐12 weeks (%)								
Moisture	79.11 ± 0.10	78.62 ± 0.17	78.19 ± 0.19	78.97 ± 0.09	78.87 ± 0.11	78.88 ± 0.08	0.339	0.465
Crude lipid	0.65 ± 0.08^b^	1.08 ± 0.03^a^	0.80 ± 0.32^b^	0.70 ± 0.04^b^	0.83 ± 0.04^b^	0.78 ± 0.06^b^	0.059	0.001
Crude protein	19.21 ± 0.15	19.34 ± 0.13	19.98 ± 0.12	19.21 ± 0.09	19.29 ± 0.10	19.26 ± 0.09	0.313	0.485

*Note:* Values are presented as means ± SEM from triplicate tanks (*n* = 6). The different letters indicate significant differences among dietary treatments as determined by one‐way ANOVA followed by Duncan’s test (*p*  < 0.05).

### 3.6. Effects of Dietary Treatments on Fatty Acid Composition of Yellow River Carp

**Figure 6:**
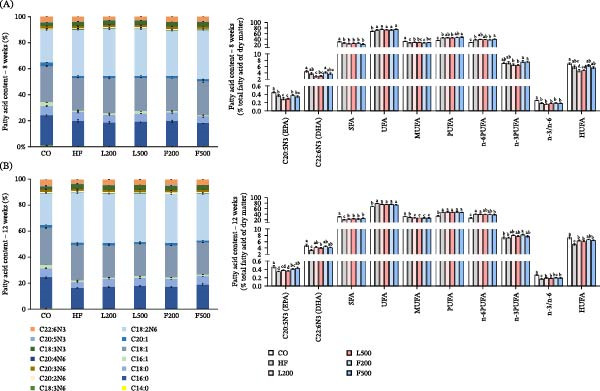
Fatty acid composition of muscle in Yellow River carp (*Cyprinus carpio*) fed experimental diets. (A, B) Fatty acid composition of muscle at 8 and 12 weeks, respectively. Values are presented as means ± SEM from triplicate tanks (*n* = 6). The different letters indicate significant differences among dietary treatments as determined by one‐way ANOVA followed by Duncan’s test (*p*  < 0.05).

The specific composition of muscle fatty acids is presented in Figure 6. At 8 weeks, the HF group exhibited significantly lower EPA, ∑SFA, MUFA, and *n*‐3/*n*‐6 ratio than the CO group, while UFA, PUFA, and total *n*‐6 PUFA were clearly elevated (*p*  < 0.05). Relative to the HF group, MUFA was obviously increased in the L200 group, and PUFA and *n*‐6 PUFA contents were clearly elevated in the F500 group (*p*  < 0.05).

At 12 weeks, the HF group had markedly lower EPA, DHA, SFA, MUFA, HUFA, and *n*‐3/*n*‐6 ratio than the CO group (*p*  < 0.05). Relative to the HF group, the L200 and L500 groups showed significantly increased DHA, SFA, HUFA, and *n*‐3/*n*‐6 ratio, whereas the F500 group showed obviously increased DHA, SFA, UFA, PUFA, *n*‐3 PUFA, HUFA, and *n*‐3/*n*‐6 ratio (*p*  < 0.05).

### 3.7. Effects of Dietary Treatments on Oxidative Status of Yellow River Carp Muscle and Hepatopancreas

**Figure 7:**
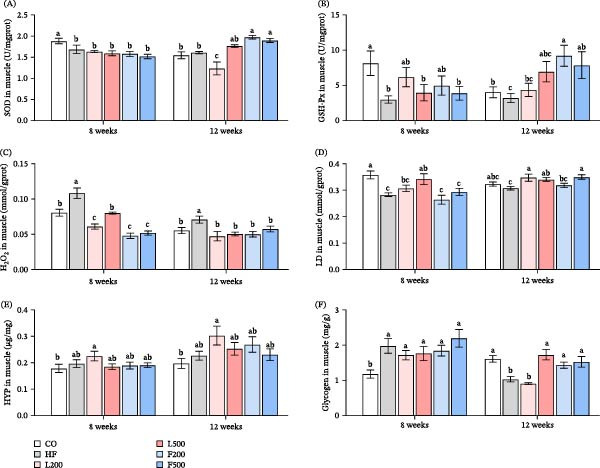
Muscle antioxidant and physicochemical parameters of Yellow River carp (*Cyprinus carpio*) fed experimental diets. (A) Superoxide dismutase (SOD). (B) Glutathione peroxidase (GSH‐Px). (C) Hydrogen peroxide (H_2_O_2_). (D) Lactic acid (LD). (E) Hydroxyproline (HYP). (F) Glycogen. Values are means ± SEM from triplicate tanks (*n* = 9) at 8 and 12 weeks. The different letters indicate significant differences among dietary treatments as determined by one‐way ANOVA followed by Duncan’s test (*p*  < 0.05).

Muscle oxidative status is presented in Figure 7. At 8 weeks, muscle H_2_O_2_ levels were substantially decreased in the L200, F200, and F500 groups compared to the HF group (*p*  < 0.05). At 12 weeks, the F200 and F500 groups had much higher SOD and GSH‐Px activities than the HF group (*p*  < 0.05).

**Figure 8:**
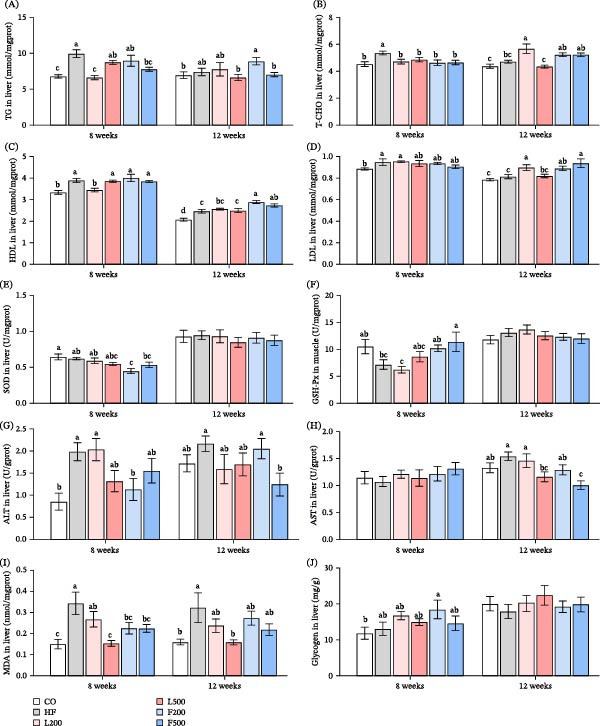
Hepatopancreatic biochemical and antioxidant parameters of Yellow River carp (*Cyprinus carpio*) fed experimental diets. (A) Triglyceride (TG). (B) Total cholesterol (T‐CHO). (C) High‐density lipoprotein (HDL). (D) Low‐density lipoprotein (LDL). (E) Superoxide dismutase (SOD). (F) Glutathione peroxidase (GSH‐Px). (G) Alanine aminotransferase (ALT). (H) Aspartate aminotransferase (AST). (I) Malondialdehyde (MDA). (J) Glycogen. Values are presented as means ± SEM from triplicate tanks (*n* = 9) at 8 and 12 weeks. The different letters indicate significant differences among dietary treatments as determined by one‐way ANOVA followed by Duncan’s test (*p*  < 0.05).

Hepatopancreatic biochemical indicators are shown in Figure 8. At 8 weeks, the TG and T‐CHO of hepatopancreatic were clearly reduced in the L200 group relative to the HF group (*p*  < 0.05), whereas ALT activity and MDA content were significantly reduced in the F200 group (*p*  < 0.05). At 12 weeks, the F500 group’s ALT and AST activities were considerably lower than the HF group (*p*  < 0.05).

### 3.8. Effects of Dietary Treatments on *gpx* Related to Lipid Metabolism, Antioxidant Defense, and Muscle Development in Yellow River Carp

The transcriptional levels of genes linked to lipid metabolism are exhibited in Figure 9. At 8 weeks, peroxisome proliferator–activated receptor γ coactivator 1‐α (*pgc-1*α), 5′‐AMP‐activated protein kinase alpha 2 (*ampkα2*), and carnitine acyl transferase 1 (*cpt-1*) were notably upregulated in the supplemented groups compared with the CO group (*p* < 0.05). Expression of the lipogenic gene fatty acid synthase (*fas*) was obviously downregulated in the F200 and F500 groups, and sterol regulatory element‐binding protein 1 (*srebp1*) was significantly downregulated in the F500 group (*p* < 0.05). In addition, lipoprotein lipase (*lpl*) was notably increased in the F200 group (*p*  < 0.05). At 12 weeks, *fas* remained significantly suppressed in all supplemented groups, and *pgc-1*α remained significantly upregulated in the L200 group (*p*  < 0.05). The F200 group exhibited considerably increased expression of *cpt-1*, *ampkα2*, and *pgc-1*α (*p*  < 0.05).

Figure 9Relative mRNA expression levels of muscle lipid metabolism–related genes in Yellow River carp (*Cyprinus carpio*) fed experimental diets. (A) *fas* (fatty acid synthase), (B) *acc* (acetyl‐coA carboxylase), (C) *srebp1* (sterol regulatory element–binding protein 1), (D) *lpl* (lipoprotein lipase), (E) *cpt-1* (carnitine acyl transferase 1), (F) *pparα* (peroxisome proliferator–activated receptor alpha), (G) *ampkα1* (5′‐AMP‐activated protein kinase alpha1), (H) *ampkα2* (5′‐AMP‐activated protein kinase alpha 2), (I) *elovl5a* (elongation of very long chain fatty acids protein 5a), (J) *elovl5b* (elongation of very long chain fatty acids protein 5b), (K) *akt* (protein kinase B), and (L) *pgc-1*α (peroxisome proliferator‐activated receptor γ coactivator 1‐α). Values are presented as means ± SEM from triplicate tanks (*n* = 9) at 8 and 12 weeks. The different letters indicate significant differences among dietary treatments as determined by one‐way ANOVA followed by Duncan’s test (*p*  < 0.05).

**Figure:**
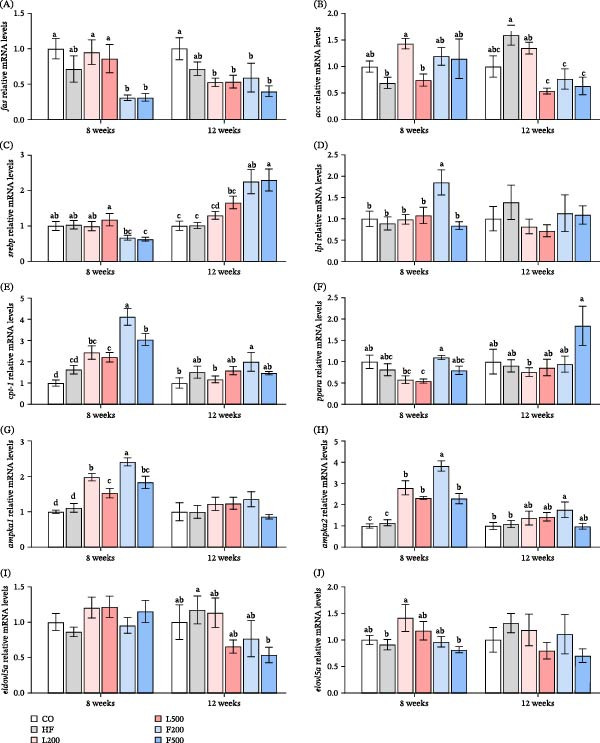

**Figure:**
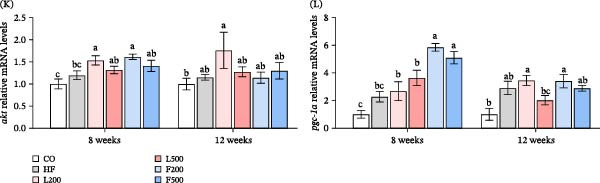

**Figure 10:**
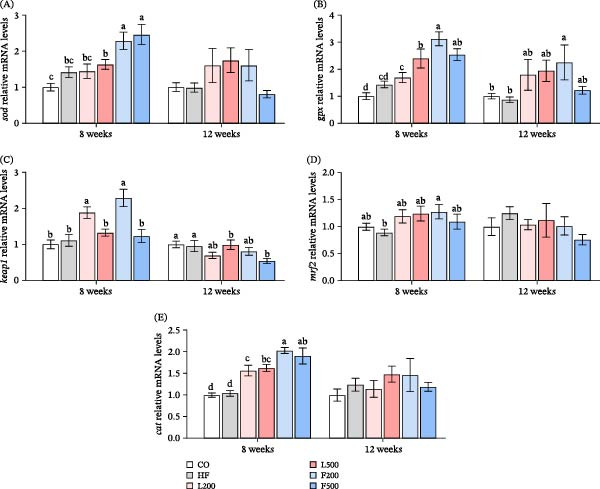
Relative mRNA expression levels of muscle antioxidant–related genes in Yellow River carp (*Cyprinus carpio*) fed experimental diets. (A) SOD (superoxide dismutase), (B) *gpx* (glutathione peroxidase), (C) *keap1* (kelch‐like ECH‐associated protein 1), (D) *nrf2* (nuclear factor erythroid 2–related factor 2), and (E) *cat* (catalase). Values are presented as means ± SEM from triplicate tanks (*n* = 9) at 8 and 12 weeks. The different letters indicate significant differences among dietary treatments as determined by one‐way ANOVA followed by Duncan’s test (*p*  < 0.05).

Antioxidant‐related *gpx* is shown in Figure 10. At 8 weeks, all supplemented groups exhibited clearly higher levels of catalase (*cat*) and GSH‐Px expression than the CO group (*p*  < 0.05). SOD expression was markedly elevated in the L500, F200, and F500 groups, while kelch‐like ECH‐associated protein 1 (*keap1*) was obviously upregulated in the L200 and F200 groups (*p*  < 0.05). At 12 weeks, *gpx* expression remained clearly higher in the F200 group (*p*  < 0.05).

**Figure 11:**
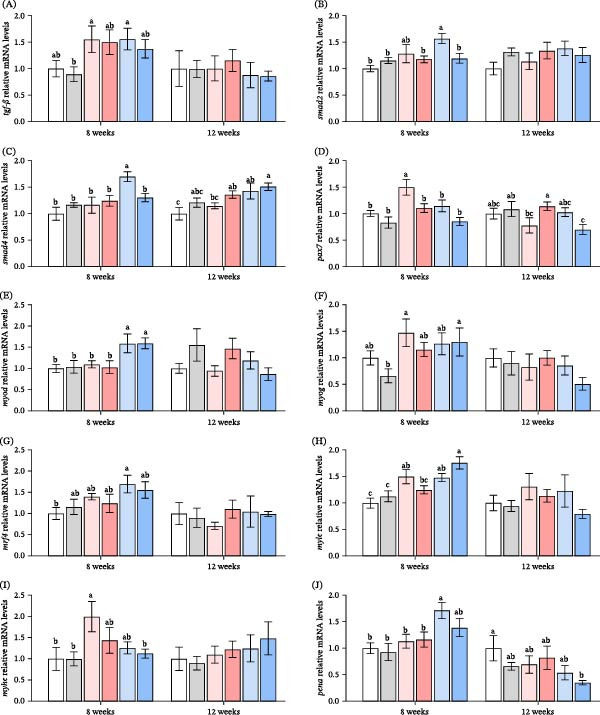
Relative mRNA expression levels of muscle genes involved in collagen synthesis and muscle development in Yellow River carp (*Cyprinus carpio*) fed experimental diets. (A) *tgf-β* (transforming growth factor‐β), (B) *smad2* (smad family member 2), (C) *smad4* (smad family member 4), (D) *pax7* (paired box 7), (E) *myod* (myogenic differentiation), (F) *myog* (myogenin), (G) *mrf4* (myogenic regulatory factors 4), (H) *mylc* (myosin light chain), (I) *myhc* (myosin heavy chain), and (J) *pcna* (proliferating cell nuclear antigen). Values are presented as means ± SEM from triplicate tanks (*n* = 9) at 8 and 12 weeks. The different letters indicate significant differences among dietary treatments as determined by one‐way ANOVA followed by Duncan’s test (*p*  < 0.05).

Genes related to collagen synthesis and muscle development are shown in Figure 11. At 8 weeks, the L200 group showed significant upregulation of transforming growth factor‐β (*tgf-β*), paired box 7 (*pax7*), myogenin (*myog*), myosin light chain (*mylc*), and myosin heavy chain (*myhc*) (*p*  < 0.05). The F200 group showed obvious upregulation of *tgf-β*, smad family member 2 (*smad2*), smad family member 4 (*smad4*), myogenic differentiation (*myod*), myogenic regulatory factors 4 (*mrf4*), *mylc*, and proliferating cell nuclear antigen (*pcna*) (*p* < 0.05). At 12 weeks, *smad4* expression in the F200 and F500 groups remained considerably higher than in the HF group (*p*  < 0.05).

## 4. Discussion

### 4.1. Short‐Term Feeding of HF Diets Promotes Rapid Growth, but Long‐Term Feeding Damages Health

Dietary lipids are indispensable macronutrients for fish because they provide concentrated energy to support both basal metabolism and somatic growth. Considering that HF diets can increase early growth performance and have a protein‐sparing effect, they are frequently used in intensive aquaculture systems [20]. In the current study, Yellow River carp fed a HF diet exhibited significantly higher FBW, WGR, and SGR during the initial feeding period, confirming that increased lipid availability can effectively support rapid growth at 8 weeks under an appropriate nutrient supply.

However, long‐term feeding did not preserve this growth advantage. As the feeding time was prolonged to 12 weeks, the HF group showed a decline in feed utilization efficiency, as evidenced by an increased FCR and excessive visceral and hepatic lipid accumulation. These findings suggest that long‐term exposure to high dietary lipid levels induces metabolic inflexibility, characterized by an impaired capacity to modify the distribution of nutrients between lipid storage and protein accretion. Similar phenomena are thought to be a significant barrier to long‐term HF feeding in aquaculture and were seen in a variety of fish species [3, 21]. Rapid short‐term growth, when accompanied by metabolic stress and lipid overload, may not always result in increased economic efficiency from a production standpoint. Therefore, dietary methods that enhance metabolic efficiency and sustain long‐term physiological stability are more desirable than those that merely maximize early growth rates.

### 4.2. L‐Carnitine and Fenofibrate Promote Energy Metabolism Rather Than Promoting Growth

Fish maintain lipid metabolic homeostasis through a dynamic balance between lipid synthesis and fatty acid oxidation. However, the current study found that HF diets exhibited significant lipid metabolic disorders at both 8 and 12 weeks, including elevated serum TG levels, increased muscle crude lipid content, and excessive lipid droplet accumulation. At 8 weeks, these lipid disorders concurrently disrupted the muscle proximate composition, as evidenced by a significant reduction in moisture and an elevation in crude protein content. Concurrently, the fatty acid composition of muscle tissue in the HF group was markedly altered, as evidenced by reduced levels of EPA, DHA, and the *n*‐3/*n*‐6 ratio, indicating that long‐term HF diets not only promoted abnormal lipid deposition but also disrupted fatty acid composition [21, 22]. Notably, at 8 weeks, the expression of fatty acid oxidation–related genes (*cpt-1* and *pgc-1*α) was upregulated, suggesting that the fish entered a metabolically active state characterized by enhanced lipid utilization. However, at 12 weeks, the expression acetyl‐coA carboxylase (*acc*) increased, while the induction of fatty acid oxidation–related genes was less pronounced than that observed at 8 weeks. Although serum TG levels, muscle lipid content, and lipid droplet accumulation in the HF group were lower at 12 weeks than at 8 weeks, they remained significantly higher than those in the CO group. Intriguingly, despite the persistent lipid deposition, muscle moisture and crude protein content showed no significant differences among all treatments at the 12‐week mark. This phenomenon likely reflects an adaptive adjustment of lipid metabolism in response to prolonged HF exposure rather than a true restoration of metabolic homeostasis. Collectively, these findings demonstrate that long‐term consumption of a HF diet progressively disrupts the lipid metabolic balance in the muscle of Yellow River carp, ultimately leading to ectopic lipid deposition within muscle tissue.

L‐carnitine and fenofibrate are widely recognized as functional feed additives that regulate lipid metabolism. Previous investigations have shown that L‐carnitine or fenofibrate promotes growth in certain fish species [23–25]; however, these effects seem to be quite species‐specific and rely on factors such as culture conditions, dietary composition, and development stage. In the current study, neither L‐carnitine nor fenofibrate significantly enhanced body weight gain in Yellow River carp. Instead, both additives markedly reduced HSI, viscerosomatic index (VSI), and IFI. This information proves that the primary role of these additives is not to directly stimulate growth but to alleviate lipid overload and improve metabolic homeostasis [25–27].

Dietary supplementation with L‐carnitine or fenofibrate effectively alleviated this metabolic disorder. At 8 weeks, the expression levels of *pgc-1*α, *ampkα2*, and *cpt-1* were significantly upregulated in all supplemented groups, *pgc-1*α and *ampkα2* are crucial biogenesis regulators in mitochondrial and energy sensing [28], with the most pronounced effect observed in the L200 and F200 groups, whereas the lipogenic genes *fas* and *srebp1* were markedly suppressed. At 12 weeks, L500 significantly decreased the *acc* expression, while F500 significantly activated PPARα expression and reduced the *acc* expression. Although the expression levels of 5′‐AMP‐activated protein kinase alpha1 (*ampkα1*), *ampkα2*, and *cpt-1* were lower than those at 8 weeks, overall muscle lipid deposition remained significantly reduced. These findings indicate that both additives can rapidly activate fatty acid oxidation pathways and inhibit lipid synthesis, thereby promoting the utilization of excess lipids. Mechanistically, L‐carnitine primarily enhances fatty acid β‐oxidation capacity [19]. In contrast, fenofibrate, as a PPARα agonist, has a broader regulatory range [17], not only significantly upregulating *cpt-1* expression but also promoting *lpl* transcription. LPL is involved in the hydrolysis of TG in circulating lipoproteins, and its increased expression suggests enhanced tissue capacity for lipid uptake and utilization [16]. Overall, metabolic regulation occurs primarily at the transcriptional level, whereas improvements in muscle lipid deposition require a longer period to become apparent.

The improvement in lipid metabolic efficiency was also reflected in favorable changes in the muscle fatty acid composition, particularly in the nutritionally important *n*‐3 long‐chain polyunsaturated fatty acids (LC‐PUFAs), EPA, and DHA, which are key determinants of fish flesh nutritional quality. At 8 weeks, although dietary supplementation had already significantly activated the AMPK and pgc‐1α signaling pathways, improvements in EPA, DHA, and total HUFAs were relatively limited. By 12 weeks, however, the L200 and L500 groups exhibited significantly higher levels of DHA, HUFAs, and the *n*‐3/*n*‐6 ratio, whereas the F200 and F500 groups further increased the *n*‐3 PUFA and total PUFA contents. EPA and DHA are HUFAs that are particularly susceptible to oxidative damage and preferential oxidation. In the present study, no sustained and significant upregulation of *elovl5* nor *fads2* was observed, suggesting that the increase in EPA and DHA was not entirely attributable to enhanced endogenous LC‐PUFA biosynthesis. Instead, the elevated EPA and DHA levels may be associated with reduced lipid deposition and improved antioxidant capacity in the supplemented fish, which could help preserve these functional fatty acids and ultimately enhance the nutritional quality of fish flesh.

### 4.3. Reduce IMF Deposits to Enhance Muscle Quality

Muscle, as the main edible part of fish, directly impacts the market value of cultured fish [29]. An appropriate IMF content is beneficial for improving flesh tenderness and juiciness, whereas excessive lipid deposition has adverse effects on muscle quality. This study found that the HF group consistently exhibited a higher IMF content and lipid droplet area for a long time. This altered the tightness of the muscle connective tissue, leading to a decrease in muscle hardness and elasticity, further reducing the fish flesh quality [30]. Similar findings have also been documented in studies of grass carp [31] and rainbow trout (*Oncorhynchus mykiss*) [32], where excessive ectopic lipid deposition is the main cause of reduced muscle hardness and quality degradation in cultured fish [32–34].

Dietary supplementation with L‐carnitine or fenofibrate significantly reduced intramuscular lipid accumulation and improved important texture parameters, including hardness, springiness, and shear force. These findings align with earlier research showing that alleviating lipid overload enhances muscle firmness and flesh quality in cultured fish [35, 36]. The results of HE staining further confirmed that L‐carnitine reduced the muscle fiber diameter while increasing fiber density. It is generally accepted that high‐quality fish flesh has this dense and slender structure of muscle fibers, indicating that altering muscle fiber properties plays a crucial role in improving meat texture.

Although both additives improved muscle quality, their underlying regulatory mechanisms differed substantially. At 8 weeks, the L200 group significantly upregulated genes associated with muscle satellite cell activation and differentiation, including *pax7*, *myog*, *myhc*, and *mylc*. Pax7 is a particular marker gene for muscle satellite cells, while myosin‐related genes (*myhc* and *mylc*) directly participate in regulating myofibrillar differentiation. Combined with the histological observations of increased muscle fiber density and reduced fiber diameter, these results suggest that L‐carnitine increases myofibril density by activating muscle satellite cell proliferation and promoting myofibril differentiation [37–40].

In contrast, fenofibrate exerted a broader effect on muscle remodeling. At 8 weeks, the F200 group showed significantly increased expression of *tgf-β*, *smad2*, and *smad4*, indicating that activation of TGF‐β/Smad signaling components in this study indicates enhanced collagen synthesis, which is critical for maintaining muscle structural integrity and improving muscle hardness [41, 42]. This pathway plays a crucial role in regulating collagen synthesis and extracellular matrix remodeling, thereby contributing to the maintenance of muscle structural integrity and mechanical strength. In addition, F200 significantly upregulated the expression of myogenic regulatory genes, including *myod*, *myog*, *mrf4*, and *mylc*, suggesting that fenofibrate not only promotes extracellular matrix remodeling but also enhances myogenic cell proliferation and differentiation. Interestingly, the responses of muscle development–related genes were primarily observed at 8 weeks, whereas differences among groups became less pronounced at 12 weeks. Nevertheless, improvements in muscle structure and texture still persisted. This finding suggests that early transcriptional changes initiate the muscle remodeling process, while long‐term changes mainly contribute to improved muscle quality.

### 4.4. L‐Carnitine and Fenofibrate Can Enhance the Antioxidant Capacity

Oxidative stress is frequently associated with excessive lipid deposition caused by a HF diet, which exacerbates oxidative damage in fish and ultimately impairs muscle quality [20]. In the present study, HF diets elevated lipid peroxidation products (MDA and H_2_O_2_) and suppressed antioxidant enzyme activities (SOD and GSH‐Px), confirming a marked decline in antioxidant defense capacity with HF loading [43].

SOD, CAT, and GSH‐Px are primarily among the antioxidant enzymes that are mostly affected by the nuclear factor erythroid 2–related factor 2 (*nrf2*)/*keap1* signaling pathway in the body. *nrf2* is a crucial transcription factor that binds to antioxidant response elements to activate the transcription of antioxidant enzyme genes. In this investigation, the F200 group raised the expression of *nrf2*, SOD, *gpx*, and *cat*, accompanied by increased GSH‐Px activity in both serum and muscle. Interestingly, F200 also elevated the *keap1* expression. Since the regulation of *keap1*‐*nrf2* mainly occurs at the protein and posttranslational levels, the rise in *keap1* mRNA might reflect a negative feedback response to oxidative stress, rather than necessarily meaning enhanced inhibition of *nrf2* activity. Instead, simultaneous upregulation of *keap1* and *nrf2* may reflect a feedback regulatory mechanism following *nrf2* activation. These findings indicate that fenofibrate may activate the antioxidant defense system and enhance the body’s ability to eliminate free radicals [44]. In addition, the L500 group exhibited the highest serum SOD activity among all treatment groups at 12 weeks, and the L500 group increased *gpx* and *cat* activity and reduced H_2_O_2_ levels, thereby enhancing free radical scavenging capacity and strengthening antioxidant capacity [45, 46]. These findings imply that L‐carnitine and fenofibrate can both alleviate oxidative damage caused by a HF diet.

## 5. Conclusion

This study evaluated whether dietary supplementation with L‐carnitine or fenofibrate could mitigate the adverse effects of a HF diet on Yellow River carp. The results showed that although a HF diet promoted growth during the early feeding stage (8 weeks), long‐term feeding for 12 weeks led to slow growth, excessive lipid deposition, increased oxidative stress, impaired muscle quality, and unfavorable alterations in muscle fatty acid composition. L‐carnitine primarily exerts its effects by enhancing fatty acid β‐oxidation, reducing fiber diameter, and increasing fiber density. Fenofibrate exhibited broader regulatory effects on lipid metabolism, antioxidant activity, and the expression of myogenic proliferation and differentiation genes. The effects of both additives were generally more pronounced at 12 weeks than at 8 weeks, indicating that prolonged nutritional intervention is required to achieve long‐term improvements in metabolic health and flesh quality. Under the conditions of the present study, 200 mg/kg was sufficient to induce significant metabolic responses, whereas 500 mg/kg generally produced greater improvements in lipid deposition, antioxidant status, and flesh quality. Additionally, future optimization of dosage and phased feeding strategies could further balance growth and quality. Therefore, both L‐carnitine and fenofibrate can be considered effective functional additives for alleviating the adverse effects of HF diets in Yellow River carp, with 500 mg/kg showing a greater overall application potential.

## Author Contributions

**Mengjuan Zhao:** writing – original draft, data curation, formal analysis, investigation. **Shaoyang Zhi**: methodology, supervision, writing – review and editing. **Chaobin Qin and Liping Yang**: writing – review and editing. **Guoxing Nie**: funding acquisition, conceptualization, supervision.

## Funding

This study was funded by the National Natural Science Foundation of China (Grants U22A20532, 32303019, and 32473180).

## Conflicts of Interest

The authors declare no conflicts of interest.

## Data Availability

According to reasonable suggestions, the corresponding author will provide information to support the research conclusion.
